# Facile Prepared MOF-OH-PAN Nanofiber for Separation Co(II) from Waste Batteries

**DOI:** 10.3390/polym16091239

**Published:** 2024-04-29

**Authors:** Cong Yin, Yang Luo, Ting Pan, Liting Ding, Chenghuang Wang, Guoyuan Yuan, Chongxiong Duan

**Affiliations:** 1 Xi’an Research Institute of Hi-Tech, Xi’an 710025, China; cyin720@163.com; 2 College of Chemistry and Chemical Engineering, Chongqing University of Science and Technology, Chongqing 401331, China; 2023205013@cqust.edu.cn (Y.L.); 2022441879@cqust.edu.cn (L.D.); wch2243768019@163.com (C.W.); 3 School of Materials Science and Energy Engineering, Foshan University, Foshan 528231, China; cechxduan@mail.scut.edu.cn

**Keywords:** MOFs, nanofiber, separation, Co(II)

## Abstract

Recovering cobalt from waste batteries is crucial for resource recycling and environmental protection. Here, MOF-OH, a Zr-based MOF, was synthesized and merged into a polyacrylonitrile (PAN) matrix to create MOF-OH-PAN nanofibers (NFs). These NFs showed a high cobalt ion adsorption capacity of 33.1 mg/g, retaining over 90% of the capacity after six cycles. The adsorption mechanism involves Co(II) surface diffusion followed by strong bonding with functional groups. This technology enables efficient cobalt recovery from waste batteries, supporting reuse and reducing resource depletion and environmental pollution. The study provides insights into waste battery resource management, highlighting environmental and economic benefits and contributing to green resource recovery and circular economy initiatives.

## 1. Introduction

The rising demand for electric vehicles in the new energy vehicle industry has underscored the significance of power batteries, specifically ternary lithium batteries, renowned for their compact size, high energy density, low-temperature resilience, and exceptional cycling capabilities. Nevertheless, the substantial utilization of cobalt resources in the production of these batteries presents a notable challenge to the industry’s sustainability and environmental conservation [1]. Recycling cobalt from discarded lithium-ion batteries can help extend the longevity of cobalt resources, diminish the need for fresh cobalt ores, and reduce the environmental impact of the new energy vehicle industry [2,3]. The manufacturing of ternary lithium batteries necessitates a substantial quantity of cobalt resources and untreated discarded lithium-ion batteries may result in cobalt loss, consequently reducing the lifespan of cobalt resources and boosting the demand for fresh cobalt ore in the production of ternary lithium batteries. Conversely, the environmental release of cobalt from discarded ternary lithium batteries poses a significant risk to both human health and the ecosystem [4]. Prolonged exposure to or ingestion of excess cobalt may induce poisoning, presenting symptoms such as headaches, vomiting, anemia, and potential adverse effects on the reproductive and immune systems. Furthermore, excessive cobalt entering the food chain can lead to environmental contamination. Ecologically, elevated cobalt levels can instigate water and soil pollution, impair plant growth, disturb the equilibrium of the animal ecosystem, and undermine the stability and biodiversity of the ecosystem. Hence, the separation and recycling of cobalt from discarded lithium-ion batteries are crucial to optimize cobalt resource utilization while mitigating its adverse effects on human health and the environment.

Numerous methods have been developed for separating pollutants in solution, with adsorption emerging as a promising industrial application method [5,6,7]. Adsorption offers several advantages, including abundant material sources, high enrichment factors, easy operation, absence of organic solvents, and material recyclability. Research has extensively focused on cobalt ion adsorption, utilizing materials such as clay minerals [8], graphene oxide [9,10], resin [3], ion-imprinted materials [11], and hybrid materials [12]. However, current research indicates that most adsorbent materials struggle to effectively separate cobalt ions, failing to meet the practical demands for cobalt separation, which require a high adsorption capacity, exceptional selectivity, and stability. Therefore, the exploitation of novel materials suitable for the industrial separation of cobalt is crucial. 

Metal-organic frameworks (MOFs) are crystalline materials, the porous structure of which is constructed by the coordination of aromatic carboxylic acids or multi-dentate organic ligands with central metal ions [13,14,15,16,17,18]. MOFs have a wide variety, abundant porosity, steerable pore size, and high surface area, making them ideal for preparing adsorbents [19,20,21,22,23]. Li et al. reported on a composite fiber material prepared through the in situ growth of Ce-based MOF on electrospun NFs using a solvothermal method. The experimental results demonstrated that the composite fibers exhibited an excellent removal efficiency towards 2,4-dichlorophenoxyacetic acid, with a maximum adsorption capacity of 200.8 mg/g. Additionally, dynamic filtration experiments revealed a removal rate of 90.3% for 2,4-dichlorophenoxyacetic acid within 6 min [14]. Wang et al. developed a high-performance thin film nanofibrous composite (TFNC) membrane consisting of a polyacrylonitrile (PAN)-UiO-66-(COOH)_2_ composite nanofibrous substrate (CPAN) and a calcium alginate (CaAlg) surface layer. The CPAN-2 substrate demonstrated exceptional adsorption capacity for Pb^2+^, with a maximum Pb^2+^ adsorption capacity of 254.5 mg/g determined using the Langmuir isotherm model. The CaAlg layer of the TFNC membrane features a porous structure, contributing to high water permeability and a dye rejection rate exceeding 95% [24]. Liu’s team conducted initial research on the separation of cobalt using functionalized MOFs [25]. They grafted Schiff base groups onto UiO-66 to create UiO-66-Schiff, cobalt ions adsorption capacity of which calculated as 256 mg/g. In our study, we doped melamine into UiO-66 using a simple method, and the adsorption capacity of the generated UiO-66-TABC for cobalt ions was 137 mg/g [26]. Furthermore, Yuan et al. prepared MOFs ion-imprinted polymers using ion imprinting technology [27], demonstrating a cobalt ion adsorpting amount of 132.8 mg/g and selective coefficients of 1.20 for Co(II)/Ni(II), 4.61 for Co(II)/Ca(II), 78.40 for Co(II)/Li(I), and 3.71 for Co(II)/K(I). Although functionalizing MOFs enhances their adsorption capacity and selectivity for cobalt ions, the complex procedures present challenges. Moreover, using MOFs as powder adsorbents in the separation and recovery processes has proven difficult. Therefore, streamlining the preparation process of MOFs and achieving efficient separation are crucial for the separation and recovery of cobalt ions. 

The electrospinning technique has emerged as a valuable method for preparing MOF NFs in recent years [28,29]. MOF NFs produced through electrospinning exhibit characteristics such as a small pore size, high porosity, and uniformity, making them well-suited for metal ions separation applications [30,31]. Some researchers have successfully incorporated MOFs into polymer matrices to enhance the efficiency of removing various metal ions from water. For instance, Yuan et al. [32] utilized UiO-66 in a polyacrylonitrile (PAN) matrix, the obtained UiO-66/PAN NFs acted as the adsorbing material to adsorb the arsenate and arsenite in water and the maximum adsorpting amount reached 42.2 mg/g and 32.9 mg/g for As(V) and As(III), respectively. In another study, Wang et al. [33] employed an in situ solvothermal method to load UiO-66 onto zirconia NFs for As(III) removal, in which the greatest As(III) adsorping amount was 143.9 mg/g. Hence, further exploration and advancement in electrospinning technology for the preparation of MOFs NFs hold significant practical importance for the effective separation of cobalt ions.

In this work, a zirconium-based MOF material, named MOF-OH, was synthesised through a one-step method, and MOF-OH-PAN NFs were synthesized through electrospinning using PAN as the matrix and MOF-OH as the filler. We will investigate the adsorption behavior of cobalt ions on these MOF-OH-PAN, studying the impacts of the pH, temperature, and initial cobalt concentration. Additionally, we will assess the recyclability of MOF-OH-PAN and explore the adsorption mechanism. Through these experiments, we aim to improve the efficiency of cobalt ion separation from discarded lithium-ion batteries using MOF NFs.

## 2. Materials and Methods

### 2.1. Chemicals and Instruments

The medicine and reagent utilized in the process of the experiment, including zirconium chloride (ZrCl_4_), 2,5-dihydroxyterephthalic acid (DHPA), and polyacrylonitrile (PAN), were purchased from Aladdin (Shanghai, China). Dimethylformamide (DMF), sodium hydroxide (NaOH), hydrochloric acid (HCl), and cobalt nitrate hexahydrate (Co(NO_3_)_2_·6H_2_O) were purchased from Chengdu Kelong Chemical Co. (Chengdu, China). All of the reagents were used directly, without any purification steps before being used.

To investigate the structure of the materials, several analytical measures were conducted, X-ray diffraction data were obtained from XRD-7000 (Shimadzu, Japan), Fourier-transform infrared spectroscopy were recorded on a Nicolet 6700 spectrometer (Thermo Fisher, Waltham, MA, USA), adsorption data obtained through Brunauer-Emmett-Teller methods were obtained through Autosorb iQ analysis (Quantachrome, Boynton Beach, FL, USA), and the scanning electron microscopy view was collected from JSM-7800F (STARJOY LIMITED, Tokyo, Japan).

### 2.2. Synthesis of MIIP

MOF-OH was prepared using the solvothermal method [33]. DHPA and ZrCl_4_, weighing 1.7 g and 2.4 g, respectively, were individually dissolved in DMF. After achieving uniform dissolution through ultrasound treatment, the two solutions were mixed together. Subsequently, 1.5 mL HCl was involved and then stirred for 1 h to mix the solution completely. The solution was then passed to a hydrothermal reactor and allowed to crystallize at a temperature of 120 °C for 1 day. After cooling the material, it was washed using DMF then dried to obtain MOF-OH.

**Scheme 1:**
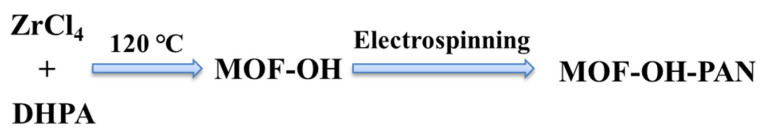
Schematic diagram of MOF-OH-PAN preparation.

Then, 10% PAN by weight was dissolved in DMF, and 5% MOF-OH was included. The solution was stirred for 6 h and then allowed to settle to remove any bubbles, resulting in the preparation of the electrospinning solution. The positive voltage was set at 12 kV, while the negative voltage was maintained at 2 kV. A metal nozzle with a 20 G specification and an injector with a solution capacity of 4 mL were used. The feed rate was set to 0.0018 mm/s, the injection distance was 15 cm, and the collecting speed was 150 r/min. Using these parameters, electrospinning was carried out to produce NFs made of MOF-OH-PAN (Scheme 1). The obtained material was then soaked and washed in deionized water at 60 °C, and finally dried for further experiments.

### 2.3. Adsorption Experiment

First, 20 mg MOF-OH-PAN was transferred to a conical flask (250 mL). Next, 50 mL solution of cobalt ion (10–50 ppm) at a precisely controlled concentration and pH (5.0–9.0) was added. The temperature of a constant temperature oscillating incubator was adjusted to the specified value (288.15–308.15 K), and the mixture was stirred at a constant shaking speed of 150 rpm for a predetermined period of time (0–9 h). After agitation, centrifugation was performed to separate the supernatant. The cobalt ion concentration data in the obtained centrifugate solution were acquired from inductively coupled plasma mass spectrometry (ICP-MS, Thermo Fisher). Blank experiments were conducted for each adsorption experiment to determine the cobalt ion concentration before and after adsorption. The adsorption capacity *q*_e_ (mg/g) can be acquired using an equation (Equation (1)) [25], in which *C*_0_ and *C*_e_ indicate concentrations (mg/L) the initial and equilibrium stage of cobalt ions, respectively; *V* indicates the volume (L) of the solution; and *m* indicates quality of the adsorbing material (g).

(1) $$q_{e}=\frac{(C_{0}-C_{e})\times V}{m}$$

## 3. Results and Discussion

### 3.1. Characterization

**Figure 1:**
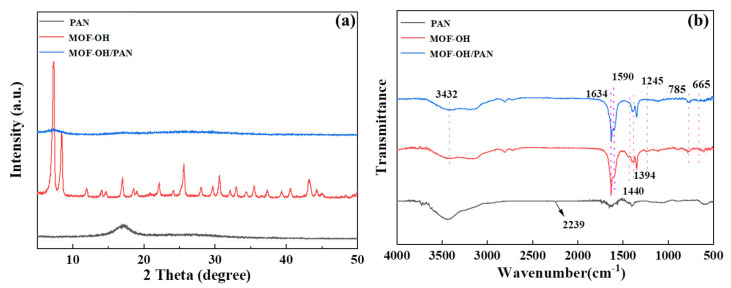
The characterization of PAN, MOF-OH, and MOF-OH-PAN involved XRD (**a**) and FT-IR (**b**) analysis.

The XRD results for MOF-OH, PAN, and MOF-OH-PAN NFs are presented in Figure 1a. The presence of diffraction peaks at 2θ = 8.8°, 12.2°, and 25.8° in pure MOF-OH particles, corresponding to the crystallographic planes of (002), (022), and (024) respectively, aligns with the characteristic XRD diffraction peaks of the typical zirconium-based MOF material UiO-66, providing evidence of the successful preparation of MOF-OH [18,22]. The identification of a peak at 2θ = 16.8° confirms the presence of the PAN polymer, offering conclusive proof of its integration into the material structure. Upon the fabrication of MOF-OH-PAN NFs with PAN as the matrix and MOF-OH as the filler, the XRD diffraction peaks of MOF-OH-PAN noticeably decreased, almost disappearing, likely due to its low content of 5% and potential complete encapsulation by PAN.

FT-IR analysis (Figure 1b) was conducted to examine the chemical structure of MOF-OH, PAN, and MOF-OH-PAN NFs. The peak at around 2239 cm^−1^ is from the C≡N stretching vibration in PAN. A broad peak at 3432 cm^−1^ was assigned as the stretching vibration of the O-H group in MOF-OH. The peak at 1590 cm^−1^ was attributed to the stretching vibration of the C=O bond [34]. Furthermore, the stretching vibration of Zr-O appeared at 665 cm^−1^ [35]. The stretching mode bands of the O-H group in MOF-OH were observed at 1245 cm^−1^ and 1440 cm^−1^. The asymmetric and symmetric stretching vibrations of the C=O bond in MOF-OH were observed at 1634 cm^−1^ [22]. From the FT-IR spectrum, the characteristic peaks belonging to MOF-OH particles and PAN polymer can both be noticed in MOF-OH-PAN NFs, indicating the successful preparation of the composite.

**Figure 2:**
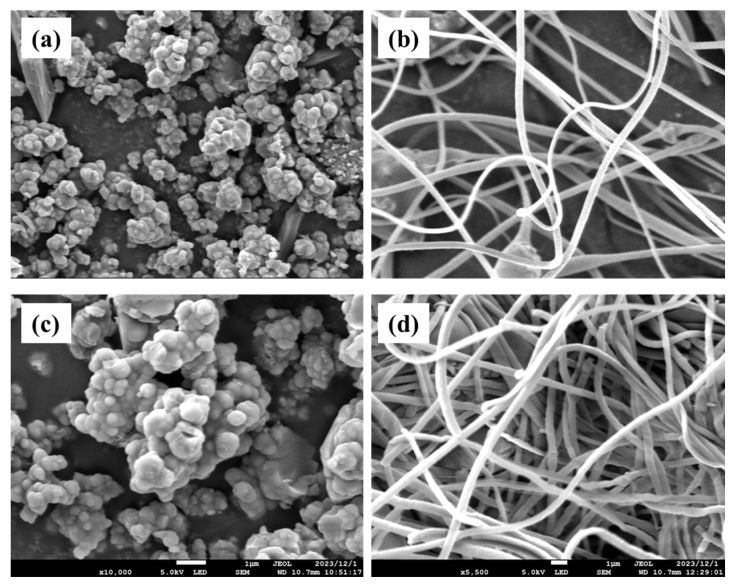
SEM of MOF-OH (**a**,**c**) and MOF-OH-PAN (**b**,**d**).

**Figure 3:**
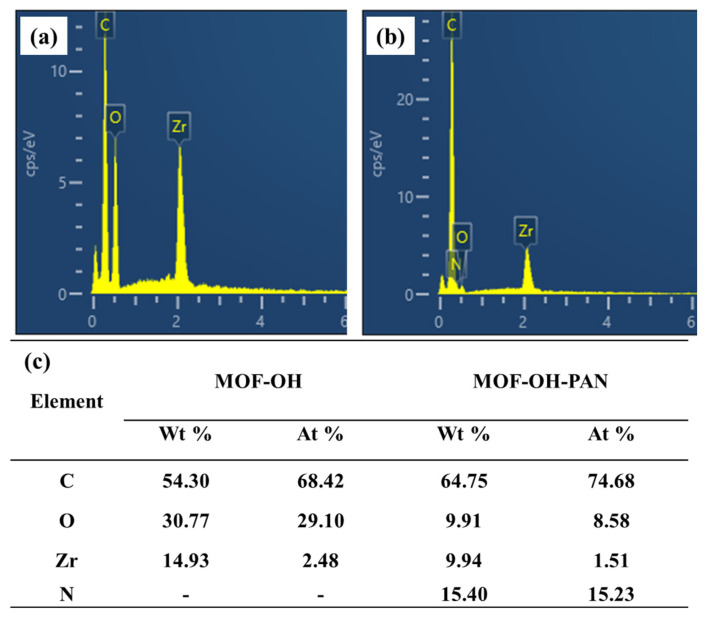
EDS analysis of MOF-OH (**a**) and MOF-OH-PAN (**b**). The elemental content of MOF-OH and MOF-OH-PAN (**c**).

From Figure 2, the morphology of MOF-OH and MOF-OH-PAN can be seen. MOF-OH appears microcrystalline, composed mainly of C, O, and Zr (Figure 3a,c). MOF-OH-PAN maintains a fibrous structure with a reduced surface smoothness after coating with MOF-OH, composed mainly of C, O, Zr, and N (Figure 3b,c).

**Figure 4:**
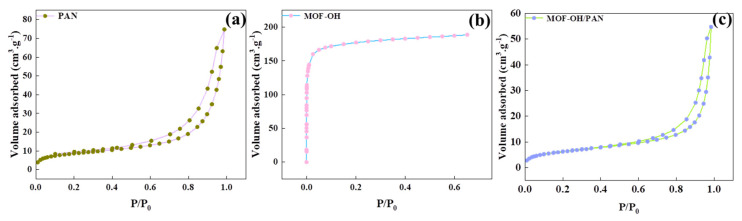
The N_2_ adsorption–desorption curves of PAN (**a**), MOF-OH (**b**), and MOF-OH-PAN (**c**).

From N_2_ adsorption-desorption curves (Figure 4) for PAN, MOF-OH, and MOF-OH-PAN, the specific surface areas are obtained and listed in Table 1. The PAN has a specific surface area of 9.89 m^2^/g, MOF-OH of 800.0 m^2^/g, and MOF-OH-PAN of 22.39 m^2^/g. This indicates the encapsulation of MOF-OH in PAN, leading to a relatively low specific surface area in MOF-OH-PAN due to the low content.

### 3.2. Adsorption Performance

#### 3.2.1. Influence of pH

**Figure 5:**
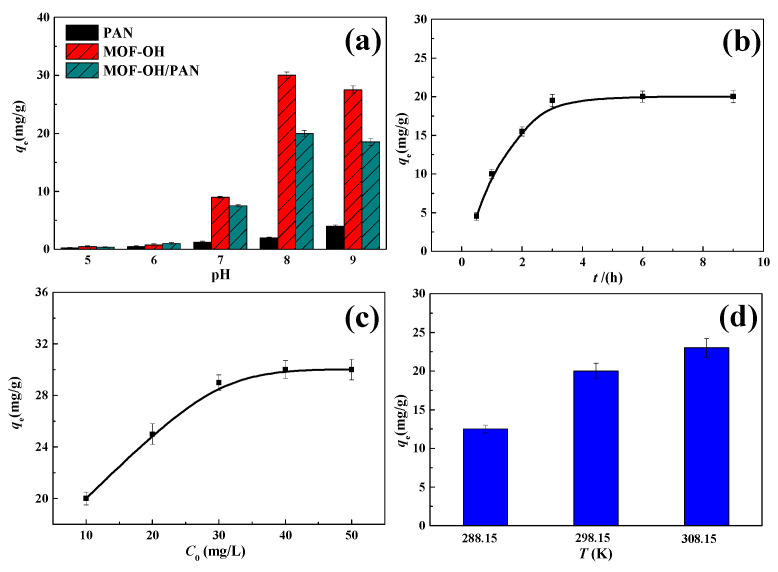
Influence of initial pH of solution (**a**), contact time (**b**), initial cobalt ion concentration (**c**), and temperature (**d**) on the adsorbent procedure. In section (**a**), the adsorbents used are PAN, MOF-OH, and MOF-OH-PAN. In sections (**b**), (**c**), and (**d**), the adsorbent used is MOF-OH-PAN. For section (**a**), *m* = 10 mg, *t* = 6 h, *T* = 298.15 K, *C*_0_ = 10 mg/L; section (**b**), *m* = 10 mg, pH = 8.0, *T* = 298.15 K, *C*_0_ = 10 mg/L; section (**c**), *m* = 10 mg, pH = 8.0, *t* = 24 h, *T* = 298.15 K; section (**d**), *m* = 10 mg, pH = 8.0, *t* = 6 h, *C*_0_ = 10 mg/L.

**Figure 6:**
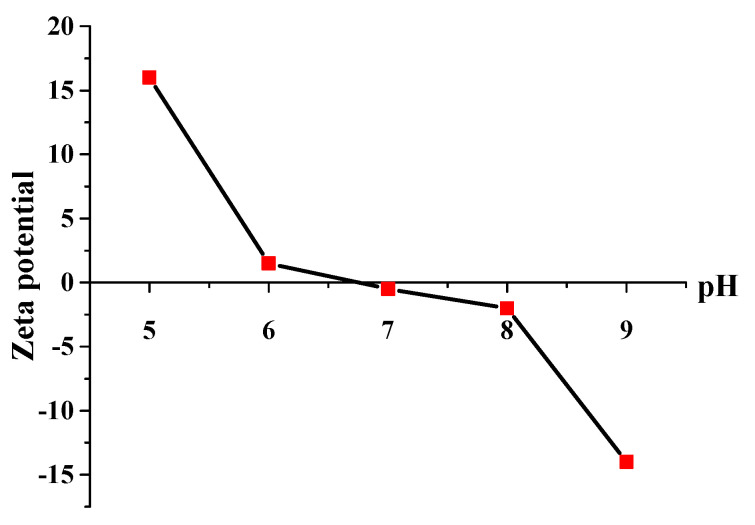
Zeta potential of MOF-OH-PAN with different pH values.

The impact of the solution pH on the adsorption property belonging to PAN, MOF-OH, and MOF-OH-PAN towards Co(II) ions is shown in Figure 5a. According to the results, the adsorption amount of these materials increases initially and then decreases when the pH level goes up from 5.0 to 9.0, with the optimal pH observed at 8.0. It is reported that cobalt ions exist in four main forms in the pH range of 5–9: Co^2+^, Co(OH)^+^, Co(OH)_2_, and Co(OH)_3_^+^. At pH 8.0, cobalt ions exist mainly in the form of Co^2+^. Under acidic conditions, the adsorption sites of the adsorbents are predominantly occupied by protonated species (Figure 6), resulting in a reduction in the available sites and diminished adsorption performance for Co(II) ions [36]. Conversely, alkaline conditions with a reduced proton concentration expose more adsorption sites, leading to an enhanced adsorption amount for cobalt ions [26]. However, at pH 9, the adsorption amount is decreased during the occurrence of the partial precipitation of cobalt ions.

Moreover, due to its multi-porous structure, higher specific surface area, and hydroxyl groups, MOF-OH exhibits a superior adsorption amount for cobalt ions compared with PAN [37]. Despite a decrease in the adsorption amount to 20 mg/g when MOF-OH is incorporated into PAN NFs, the doping level of MOFs within NFs remains at only 5%, significantly enhancing the utilization efficiency of MOFs. Therefore, forthcoming studies will be directed towards a more in-depth analysis of the adsorption capabilities of MOF-OH-PAN.

#### 3.2.2. Influence of Contact Time

In Figure 5b, the relationship between the adsorption efficiency of MOF-OH-PAN for cobalt ions and the contact time is studied. The findings demonstrate that the adsorption process achieves nearly equilibrium status in just 3 h, indicating its rapid nature. To explore the adsorption mechanism, pseudo-first-order (PFO) and pseudo-second-order (PSO) kinetic models [24,26,35,37] are examined as Equations (2) and (3), respectively. The equations for the two models are as follows:

(2) $$\ln(q_{e}-q_{t})=\ln q_{e}-k_{1}t$$

(3) $$\frac{t}{q_{t}}=\frac{1}{k_{2}{q_{e}}^{2}}+\frac{t}{q_{e}}$$

In this context, *q_t_* and *q_e_* designate the amount of adsorption at time *t* and at equilibrium (mg/g), respectively. Meanwhile, *k*_1_ and *k*_2_ represent the rate constants in PFO and PSO kinetic models, respectively. 

**Figure 7:**
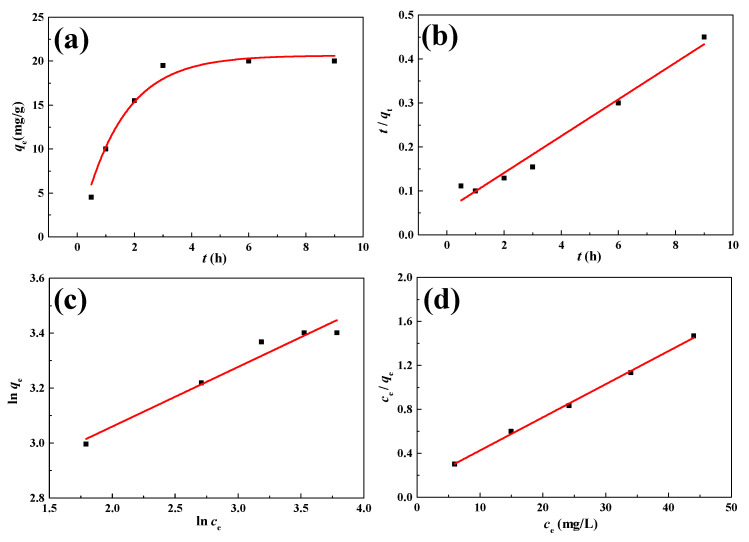
The kinetic models (PFO (**a**) and PSO (**b**)) and the isotherm models (Langmuir (**c**) and Freundlich (**d**)) for Co(II) adsorption on MOF-OH-PAN.

The fitting results of kinetic models are illustrated in Figure 7a,b. The fitting result is displayed in Table 2. Both models exhibit high correlation coefficients, indicating the adsorpting process of MOF-OH-PAN for Co(II) involves two kinetic processes: surface diffusion and chemical bonding. This suggests that Co(II) initially undergoes surface diffusion from the solution to the adsorbent’s surface, followed by strong bonding with the functional groups of MOF-OH-PAN through chemical bonding.

#### 3.2.3. Influence of Concentration

The relationship between adsorption amount and the initial concentration was examined through altering the initial concentration of cobalt ions, as illustrated in Figure 5c. The observation revealed that the adsorbing amount of Co(II) onto MOF-OH-PAN was enhanced from 20.0 mg/g to 30.0 mg/g as the original concentration of Co(II) was raised from 10 mg/L to 30 mg/L. Nevertheless, once the original Co(II) concentration enhanced to 50 mg/L, MOF-OH-PAN reached maximum adsorbing amount for Co(II), indicating that all of the adsorption sites had been tied up and reached equilibrium. Adsorption isotherms offer essential data regarding the quantity of adsorption and potential adsorption modes within the adsorption system. Therefore, Langmuir and Freundlich adsorption isotherm models [38,39,40] were evaluated in this study, which were calculated following Equations (4) and (5), respectively:

(4) $$\frac{c_{e}}{q_{e}}=\frac{c_{e}}{q_{m}}+\frac{1}{q_{m}K_{L}}$$

(5) $$\ln q_{e}=\ln K_{F}+\frac{1}{n}\ln c_{e}$$

where *q_m_* presents the theoretical maximum adsorption amount in mg/g, *C_e_* corresponds to the Co(II) concentration (mg/L) in the state of equilibrium, *K_L_* respents the Langmuir adsorption equilibrium constant, and *K_F_* and *n* are both Freundlich constants affected by the adsorping amount and intensity, respectively.

Figure 7c,d presents the fitting curves of the isotherm models. The corresponding data are displayed in Table 3, indicating that the correlation coefficient (R^2^) for the Langmuir isotherm model is 0.998, and for the Freundlich isotherm model, the R^2^ value is 0.958. The higher R^2^ value indicates a more accurate data fitting, implying the adsorpting process of MOF-OH-PAN for Co(II) by follows a uniform monolayer adsorption.

#### 3.2.4. Influence of Temperature

Figure 5d showcases how temperature impacts the adsorption of MOF-OH-PAN. The adsorpting amount of MOF-OH-PAN rises as the temperature increases, in which the amount increases from 12.5 mg/g at 288 K to 23.0 mg/g at 308 K. Above phenomenon demonstrates that higher temperatures are beneficial for the adsorpting process. Thermodynamic equations (Equations (6) and (7)) were employed to calculate the thermodynamic parameters, which include the enthalpy change (∆*H*^0^, kJ/mol), entropy change (∆*S*^0^, J/mol K), and Gibbs free energy change (∆*G*^0^, kJ/mol) [41,42].

(6) $$\ln K_{d}=\frac{\Delta S^{0}}{R}-\frac{\Delta H^{0}}{RT}$$

(7) $$\Delta G^{0}=\Delta H^{0}-T\Delta S^{0}$$

Here, *K* represents the thermodynamic equilibrium constant and *T* represents the value of temperature in Kelvin. R is the ideal gas constant (J/mol K), the value of which is 8.314. In Table 4, the calculated results of thermodynamic parameters are displayed. The positive ∆*H*^0^ value of MOF-OH-PAN demonstrates that the adsorpting procedure is endothermic, which aligns with the higher adsorption amount observed at elevated temperatures. Therefore, a higher temperature is conductive to the Co(II) adsorpting process of MOF-OH-PAN. The fact that ∆*G*^0^ is negative confirms the adsorpting process is spontaneous. Moreover, the more negative the ∆*G*^0^ value is at higher temperatures, the more favorable it is for the adsorption reaction.

#### 3.2.5. Reusability and Stability

**Figure 8:**
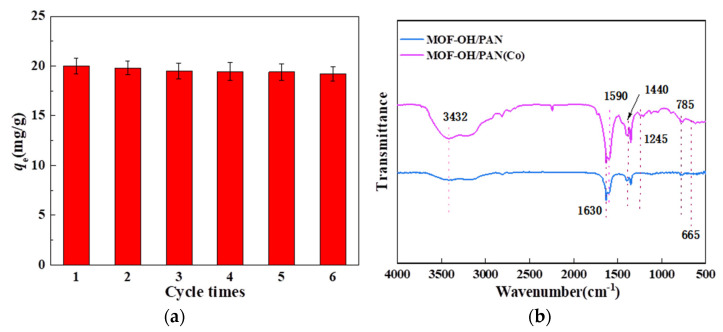
Cyclic stability test of MOF-OH-PAN (**a**) and FT-IR analysis of MOF-OH-PAN before and after adsorption (**b**). The eluent solvent is 0.1 mol/l HNO_3_. The adsorption conditions: *m* = 10 mg, *t* = 6 h, *T* = 298.15 K, *C*_0_ = 10 mg/L, pH = 8.0.

The reusability performance is an essential indicator in assessing the potential for adsorbent reuse. It significantly influences the adsorption efficiency, adsorption amount, and cost when using adsorbents for wastewater treatment. Figure 8a shows the adsorpting property and FT-IR spectrum of MOF-OH-PAN for Co(II) removal after six cycles of adsorption–desorption, indicating that the nanofiber material maintains a high removal rate and stability even after multiple cycles. The material’s high stability and reusability are showcased, making it ideal for removing cobalt ions from wastewater and offering valuable guidance for treating cobalt-containing wastewater. 

#### 3.2.6. Adsorption Mechanism

**Figure 9:**
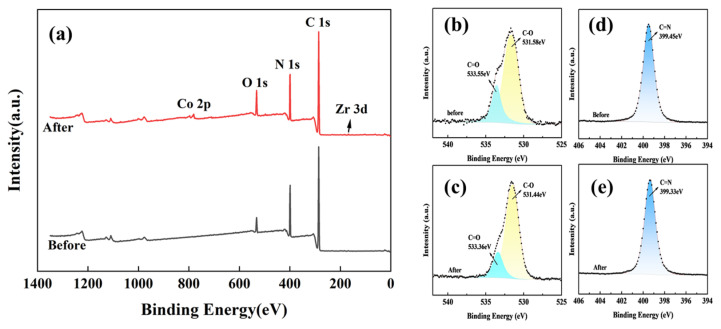
The XPS spectra of MOF-OH-PAN before and after the adsorption process. (**a**) XPS scan full spectrum, (**b**,**c**) O 1 s spectrum, and (**d**,**e**) is the N 1 s spectrum.

The adsorption process of Co(II) by MOF-OH-PAN was thoroughly examined using X-ray Photoelectron Spectroscopy (XPS) analysis. Figure 9 illustrates the XPS full spectrum, as well as the O 1 s and N 1 s fitting spectra of MOF-OH-PAN both before and after adsorption.

Analysis of the XPS full spectrum showed the emergence of the Co 2p peak, providing clear evidence of successful Co(II) adsorption by MOF-OH-PAN. The O 1 s XPS spectra of MOF-OH-PAN prior to adsorption displayed peaks at 533.55 eV and 531.58 eV, representing carbonyl oxygen and hydroxyl oxygen, respectively. Post adsorption, the peak at 533.55 eV shifted to 533.36 eV, while the peak at 531.58 eV shifted to 531.44 eV, indicating an interaction between the oxygen atoms on the ligand and Co(II).

Upon examination of the N 1 s spectra, changes in binding energy before (399.45 eV) and after adsorption (399.33 eV) suggested that nitrogen atoms also played a role in the adsorption process. These results from the XPS analysis demonstrate the coordination mechanism of Co(II) bonding with oxygen and nitrogen atoms during the adsorption process. 

## 4. Conclusions

The article presents the preparation and characterization of MOF-OH-PAN NFs to separate Co(II) from waste power batteries. The maximum adsorpting amount of Co(II) on MOF-OH-PAN was measured to be 33.1 mg/g at pH 8.0 in 25 °C. Co(II) adsorption was determined as a spontaneous and endothermic procedure, with chemical adsorption as the main rate-controlling step. The Langmuir isothermal adsorption model was utilized to account for the monolayer adsorption of Co(II) on MOF-OH-PAN. The material also exhibited a good cyclic stability, indicating its potential for cobalt separation from waste power batteries. Overall, MOF-OH-PAN shows promise as a viable option for this application.

## Figures and Tables

**Table 1:** The N_2_ adsorption–desorption isotherms of PAN, MOF-OH, and MOF-OH-PAN.

Sample	Specific Surface Area (m^2^/g)	Pore Volume (cc/g)	Pore Size (nm)
PAN	9.89	0.026	10.41
MOF-OH	800.0	0.295	1.31
MOF-OH-PAN	22.39	0.083	17.64

**Table 2:** The kinetic parameters of MOF-OH-PAN for Co(II) adsorption.

PFO			PSO		
*q*_e_	*k*_1_	R^2^	*q*_e_	*k*_2_	R^2^
20.6	0.681	0.976	23.8	0.031	0.975

**Table 3:** The parameters in the model of Langmuir and Freundlich isotherm.

Langmuir			Freundlich		
*q_m_*	*K_L_*	R^2^	ln*K_F_*	*n*	R^2^
33.1	0.246	0.998	2.63	3.69	0.958

**Table 4:** Thermodynamic parameters for adsorption of MOF-OH-PAN for Co(II).

Δ*G*^0^ (kJ/mol)			Δ*H*^0^ (kJ/mol)	Δ*S*^0^ (J∙mol^−1^∙K^−1^)
288 K	298 K	308 K		
−1.39	−2.65	−3.90	34.8	125.6

## Data Availability

Data are contained within the article.
